# Identification of the *UBP1* Locus as a Critical Blood Pressure Determinant Using a Combination of Mouse and Human Genetics

**DOI:** 10.1371/journal.pgen.1000591

**Published:** 2009-08-07

**Authors:** Hana Koutnikova, Markku Laakso, Lu Lu, Roy Combe, Jussi Paananen, Teemu Kuulasmaa, Johanna Kuusisto, Hans-Ulrich Häring, Torben Hansen, Oluf Pedersen, Ulf Smith, Markolf Hanefeld, Robert W. Williams, Johan Auwerx

**Affiliations:** 1 Institut Clinique de la Souris, Illkirch, France; 2 Department of Medicine, University of Kuopio, Kuopio, Finland; 3 Center for Integrative and Translational Genomics, University of Tennessee Health Science Center, Memphis, Tennessee, United States of America; 4 Department of Internal Medicine, Division of Endocrinology, Diabetology, Vascular Medicine, Nephrology, and Clinical Chemistry, University of Tübingen, Tübingen, Germany; 5 Hagedorn Research Institute, Copenhagen, Denmark; 6 Faculty of Health Science, University of Southern Denmark, Odense, Denmark; 7 Faculty of Health Science, University of Aarhus, Aarhus, Denmark; 8 Institute of Biomedical Science, University of Copenhagen, Copenhagen, Denmark; 9 The Lundberg Laboratory for Diabetes Research, Center of Excellence for Cardiovascular and Metabolic Research, Department of Molecular and Clinical Medicine/Diabetes, The Sahlgrenska Academy at Göteborg University, Göteborg, Sweden; 10 Centre for Clinical Studies, GWT-TUD GmbH, Dresden, Germany; 11 Faculté de Medicine, Université Louis Pasteur, Strasbourg, France; 12 Laboratory of Integrative and Systems Physiology (LISP), Ecole Polytechnique Fédérale de Lausanne, Lausanne, Switzerland; Stanford University School of Medicine, United States of America

## Abstract

Hypertension is a major health problem of largely unknown genetic origins. To identify new genes responsible for hypertension, genetic analysis of recombinant inbred strains of mice followed by human association studies might prove powerful and was exploited in our current study. Using a set of 27 recombinant BXD strains of mice we identified a quantitative trait locus (QTL) for blood pressure (BP) on distal chromosome 9. The association analysis of markers encompassing the syntenic region on human chromosome 3 gave in an additive genetic model the strongest association for rs17030583 C/T and rs2291897 G/A, located within the UBP1 locus, with systolic and diastolic BP (rs17030583: 1.3±0.4 mmHg p<0.001, 0.8±0.3 mmHg p = 0.006, respectively and rs2291897: 1.5±0.4 mmHg p<0.001, 0.8±0.3 mmHg p = 0.003, respectively) in three separate studies. Our study, which underscores the marked complementarities of mouse and human genetic approaches, identifies the UBP1 locus as a critical blood pressure determinant. UBP1 plays a role in cholesterol and steroid metabolism via the transcriptional activation of *CYP11A*, the rate-limiting enzyme in pregnenolone and aldosterone biosynthesis. We suggest that UBP1 and its functional partners are components of a network controlling blood pressure.

## Author Summary

To identify new genes responsible for hypertension, a major health problem, we performed a genetic analysis of recombinant inbred strains of mice followed by human association studies. Using a set of 27 recombinant BXD strains of mice, we identified a quantitative trait locus for blood pressure (BP) on distal chromosome 9 encompassing the UBP1 gene locus. In three subsequent human genetic studies, the syntenic locus was shown to be associated with systolic and diastolic BP, identifying the UBP1 locus as a critical blood pressure determinant. UBP1 is a transcription factor, known to control the expression of *CYP11A*, the rate-limiting enzyme in pregnenolone and aldosterone biosynthesis, suggesting that it controls blood pressure via this pathway. Our study furthermore underscores the marked complementarities of mouse and human genetic approaches

## Introduction

Elevated arterial blood pressure is a major health problem accounting for a large proportion of cardiovascular morbidity and mortality world-wide. It is estimated that over one billion people are hypertensive, and its prevalence is continuously increasing [1]. Elevated BP is a major risk factor for the development of cardiovascular disease, including coronary heart disease, congestive heart failure, ischemic and hemorrhagic stroke, renal failure, and peripheral arterial disease [2]. BP is modulated by both genetic and environmental factors, including NaCl intake, alcohol consumption, physical inactivity, and stress. Several genes responsible for rare Mendelian forms of hypertension have been identified (*CYP11B1*, *CYP11B2*, *HSD11B1*, *MR*, *SCNN1B*, *SCNN1G*, *WNK1*, *WNK4*) and shown to play a significant role in the renal control of BP [3]–[9]. In addition, rare independent mutations in salt handling genes *SLC2A3*, *SLC12A1* and *KCNJ1* have been shown to reduce BP and protect from the development of hypertension [10]. However, common genetic variants that are associated with BP remain to be identified.

Using a set of recombinant BXD strains of mice we identified a quantitative trait locus for blood pressure. To validate the role of this QTL in blood pressure control in man we used an association analysis of markers encompassing the syntenic region in humans and confirmed *UBP1* locus as a critical blood pressure determinant.

## Results

We have exploited a genetic reference panel of recombinant inbred strains of mice to map chromosomal regions that influence blood pressure under tightly controlled baseline laboratory conditions. We measured systolic BP in a panel of 27 male and 21 female BXD recombinant inbred mouse strains [11], all from the new series of strains (BXD43 through BXD103). These strains have all been genotyped using a high density panel of single nucleotide polymorphisms (SNPs) [11] and this set can provide up to twice the mapping resolution of an equal number of conventional recombinant inbred strains.

**Figure 1:**
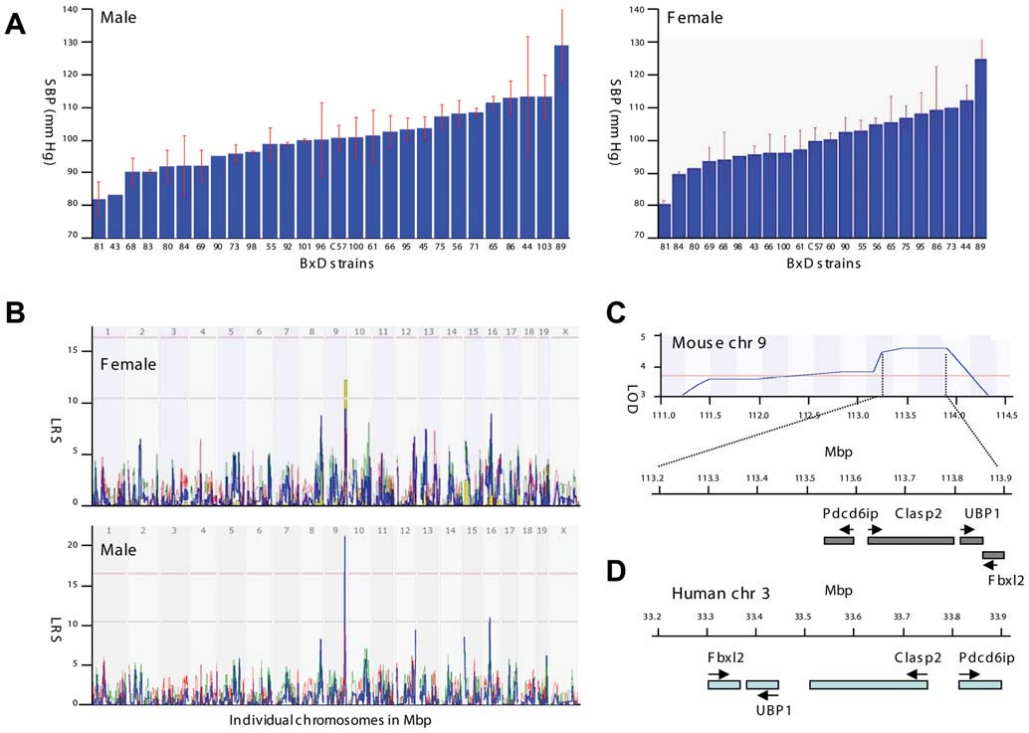
Mapping of a systolic blood pressure (SBP) quantitative trait locus (QTL) in BXD strains. (A) Rank ordered mean BP values across 27 male and 21 female BXD strains (with SEM error bars). (B) In males, a significant BP QTL is mapped on chromosome 9. In females, BP QTL maps to the same region on chromosome 9. (C) The genomic region corresponding to the BP QTL on chromosome 9. The LOD score is depicted in blue with the highest values at the position 113.2 to 113.9 Mb achieving LOD of 4.7. Individual genes under the BP QTL peak are indicated. (D) The syntenic chromosomal region in human.

BP varied widely across BXD strains (Figure 1A); from a low of 81.7±5.4 mmHg to a high of 128.3±11.4 mmHg. These strain differences were common to both sexes and the correlation between sexes was 0.86. We performed a QTL mapping of systolic BP using GeneNetwork (www.genenetwork.org, see BXD phenotype database IDs 11017 and 12076 for males and females, respectively). A significant QTL modulating BP was detected on Chr 9 between approximately 111.0 and 114.2 Mb (2-LOD CI, genome-wide p<.05), with a peak likelihood ratio statistics (LRS) of 21.6 (LOD = 4.7) in the interval between 113.2 and 113.9 Mb in the males (Figure 1B and 1C). The same interval also generated the highest genome-wide linkage in the smaller female sample (n = 21 strains), and the LRS was more modest.

The mouse BP QTL covers a genomic region of unusually low gene density (Figure 1C) that includes the *Pdcd6ip*, *Clasp2*, *Ubp1*, and *Fbxl2* genes within the QTL. PDCD6IP is a ubiquitously expressed protein that plays a role in apoptosis, virus budding, multivesicular bodies cargo sorting and cell surface receptor down regulation [12]–[16]. PDCD6IP interacts with vasopressin receptor V2R and when overexpressed, increases the lysosomal degradation of the vasopressin receptor [12]. *Clasp2* is the second candidate gene. It is microtubule plus-end tracking protein that plays a role in microtubule dynamics [17]. At present, no link exists between *CLASP2* function and BP. *Ubp1*, also termed *LBP1-a* or *NF2D9*, is the third candidate gene within the BP QTL. It is an ubiquitously expressed member of the grainyhead transcription factor family [18]. The null allele of the mouse *Ubp1* gene is lethal during early embryogenesis due to placenta insufficiency attributable to a defect in extraembryonic angiogenesis [19]. The last candidate gene, *FBXL2* belongs to the FBL family of proteins that facilitate ubiquitination of the substrate proteins.

**Figure 2:**
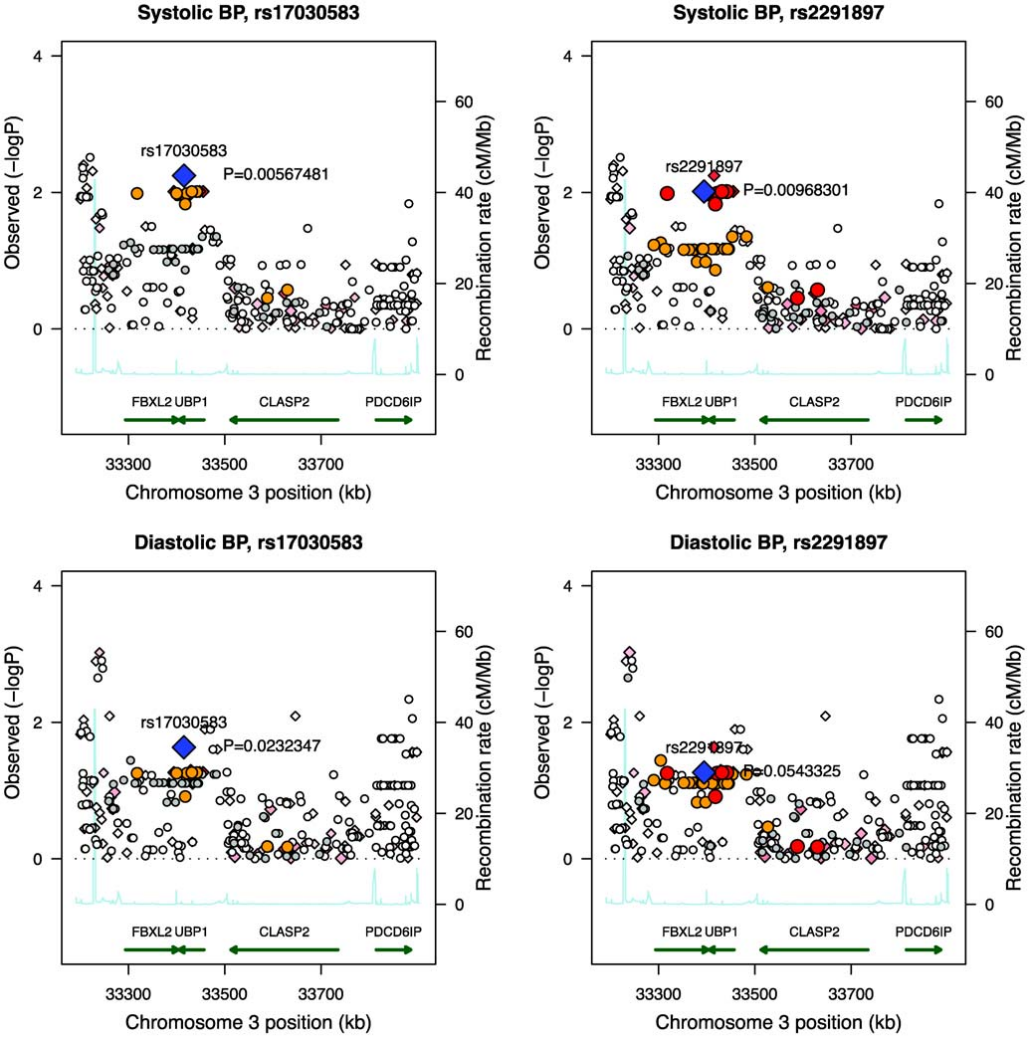
Regional association plots of the genomic location indicated by the mouse model in the EUGENE2 human study population. Associations of SNPs rs17030583 and rs2291897 (blue diamonds) to systolic and diastolic blood pressure are plotted with their P-values (as −log10 values) as a function of genomic position (with NCBI build 36). Estimated recombination rates (from Hapmap Phase 3) are plotted to reflect the local LD structure around the associated SNPs and their correlated proxies (red: r^2^≥0.8; orange: 0.5≤r^2^<0.8; gray: 0.2≤r^2^<0.5; white: r^2^<0.2). Diamonds represent directly genotyped markers and circles represent imputed markers.

To efficiently evaluate the strength of these four candidates we opted to study single nucleotide polymorphisms in human cohorts for which excellent data on BP are already available as part of the EUGENE2 cohort (N = 867) [20]. Initial marker screening was directed to the genomic location indicated by the analysis of the BXD mouse lines (human chromosome 3, 33.2–33.9 Mb region). A total of 290 markers were located in this region (73 SNPs directly genotyped, 217 imputed). Association analysis for both systolic BP and diastolic BP was performed for these markers. Two SNPs in the region of *UBP1-FBXL2* gave the strongest association (rs17030583, *UBP1*, intron 11, C/T; rs2291897, *FBXL2*, intron 11, G/A; Figure 2, Table 1). We also analyzed the results in 788 participants of the EUGENE2 study without antihypertensive treatment. The associations of rs17030583 and rs2291897 with SBP (*P* 0.010 and 0.011, respectively) and DBP (*P* 0.069 and 0.172, respectively) remained essentially similar as in the entire EUGENE2 cohort given the smaller sample size. The associations with these SNPs were replicated in two other cohorts, the Finnish METSIM (Metabolic Syndrome In Men) (N = 2537) [21] and the German RIAD (Risk factors in Impaired glucose tolerance for Atherosclerosis and Diabetes) (N = 237) (Table 1) [22]. Since the age range of subjects in the EUGENE2 study was ≤55 years, we restricted statistical analyses of the replication cohorts also to the same age category.

**Table 1:** The effects of two SNPs of the *UBP1* gene locus on systolic and diastolic blood pressure (BP. mean±SD or SE) in three different Caucasian cohorts under the additive model.

SNP ID	Cohort	N	Minor allele frequency	BP. (mmHg)*	Effect size. (mmHg) (SE)	P value
**Systolic BP**						
rs17030583	EUGENE2	867	0.24	123 (17)	+2.4 (0.9)	0.006
	METSIM	2532	0.3	137 (18)	+0.8 (0.5)	0.076
	RIAD	237	0.26	131 (17)	+3.5 (1.8)	0.063
	**Pooled**	**3636**	**0.29**		**+1.3 (0.4)**	**0.00099**
rs2291897	EUGENE2	866	0.2	123 (17)	+2.8 (0.9)	0.01
	METSIM	2537	0.27	137 (18)	+1.0 (0.5)	0.028
	RIAD	237	0.21	132 (18)	+2.2 (2.1)	0.297
	**Pooled**	**3640**	**0.25**		**+1.5 (0.4)**	**0.00036**
**Diastolic BP**						
rs17030583	EUGENE2	867	0.24	78 (12)	+2.1 (0.6)	0.023
	METSIM	2532	0.3	90 (11)	+0.4 (0.3)	0.136
	RIAD	237	0.26	83 (11)	+0.9 (1.0)	0.452
	**Pooled**	**3636**	**0.29**		**+0.8 (0.3)**	**0.0063**
rs2291897	EUGENE2	866	0.2	78 (12)	+2.0 (0.7)	0.056
	METSIM	2537	0.27	90 (11)	+0.6 (0.3)	0.051
	RIAD	237	0.21	83 (11)	+0.7 (1.2)	0.55
	**Pooled**	**3640**	**0.25**		**+0.8 (0.3)**	**0.003**

*: Systolic and diastolic BP (SD) for the common homozygote.

We observed statistically significant associations of systolic and diastolic BP with these two SNPs under the additive model (Table 1). SNP rs2291897 (G/A) in the pooled data was significantly associated with systolic (*P* = 0.00036) and diastolic BP (*P* = 0.0030). The pooled effect size of this SNP was 1.5 (0.4) for systolic and 0.8 (0.3) mmHg with diastolic BP. Similarly, rs17030538 (C/T) was associated with systolic (*P* = 0.00099) and diastolic BP (*P* = 0.00036), with pooled effect size of 1.3 (0.4) for systolic and 0.8 (0.3) mmHg with diastolic BP. We also determined the association of rs17030583 and rs2291897 with BP levels in the entire cohort of the METSIM Study (N = 7422) (rs17030583, *P* values of 0.510 and 0.773 for an association with systolic and diastolic BP, respectively; rs2291897, *P* values of 0.333 and 0.362 for an association with systolic and diastolic BP, respectively) and RIAD Study (N = 617) (rs17030583, *P* values of 0.107 and 0.191 for an association with systolic and diastolic BP, respectively; rs2291897, *P* values of 0.420 and 0.049 for an association with systolic and diastolic BP, respectively), and found no association with BP levels. The lack of association was limited to the age range >55 years, and was explained by a very strong interaction of rs17030583 and rs2291897 with age on their effects on systolic BP (rs2291897: METSIM Study *P* = 6.4×10^−87^, RIAD Study *P* = 7.3×10^−4^).

## Discussion

We have mapped an interval on chromosome 9 between 113.2 and 113.9 Mb as contributing to the BP variation in a BXD panel of mice. The relatively small size of this chromosomal region can be explained by a high frequency of recombination achieved in the recombinant inbred mouse lines.

The identified mouse chromosome 9 band F3 (9F3) region is syntenic to rat chromosome 8q32 to which a BP QTL was previously mapped [23]. The corresponding human chromosome band is 3p22.3, remarkably enough also within a known susceptibility locus for arterial pulse pressure—the difference between systolic BP and diastolic BP [24]. These data indicate that in three species an orthologous region modulates BP.

**Figure 3:**
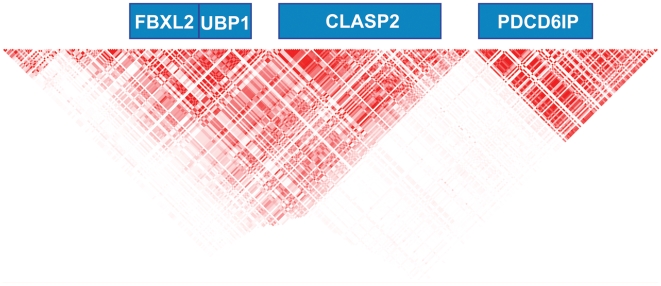
Gene and LD block structure of the human chromosome 3. 33.2–33.9 Mb region. Based on the imputed EUGENE2 data. Dark red color represents perfect LD (R^2^) of 1 between the markers.

Our SNP analysis of the syntenic region in humans (human chromosome 3, 33.2–33.9 Mb region) revealed that rs17030583, *UBP1*, intron 11, C/T and rs2291897, *FBXL2*, intron 11, G/A gave the most significant P values for an association with BP levels under the additive model in the EUGENE2 Study. The *FBXL2/UBP1* gene locus (Figure 3) belongs to a haploblock including also other genes (*CLASP2*, *PDC61P*, *SUSD5*). Therefore, we genotyped in the METSIM cohort those SNPs of *CLASP2* (rs9841066), *PDC61P* (rs9311032, rs9858195), and *SUSD5* (rs4678778, rs9836433, rs10222597) which gave the most significant P values in the EUGENE2 Study. We did not find any significant associations of these SNPs either with systolic or diastolic BPs in the METSIM Study suggesting that the *FBXL2/UBP1* locus represents the best association signal with systolic and diastolic BPs from that gene region. SNP rs2291897 is located on Chr 3 (position 33394426) in intron 11 of *FBXL2*, and is almost in complete linkage disequilibrium (99.6%) with rs2272152 located 23 bp from the acceptor site of exon 4 of *UBP1*. Because we also found a significant association of rs17030583 of *UBP1* with systolic BP it is likely that our findings in the haploblock are explained by a signal from the *FBXL2/UBP1* region (linkage disequilibrium between rs2291897 and rs17030583 was 75% in the EUGENE2 Study). However, we cannot rule out the possibility that our findings are explained by another gene locus at linkage disequilibrium with the *FBXL2/UBP1* region. It remains to be determined whether these SNPs are replicated in other population-based studies including individuals ≤55 years and whether these SNPs have functional consequences on *UBP1* splicing and/or mRNA generation. Two recent meta-analyses did not report an association of these SNPs with high systolic or diastolic BPs [25],[26], which might be due to higher age range when compared to our study (<70 years in [25]; <85 years in [26]). We found a strong interaction of rs17030583 and rs2291897 with age on their effects on systolic BP, which might indicate that these associations are observed only in middle-aged and younger individuals.

*UBP1* regulates expression of *CYP11A*, the gene encoding the rate-limiting enzyme in steroidogenesis, and is expressed in placenta and adult adrenals [27]. An adrenal steroid biosynthesis alteration via CYP11A and the 3β-HSD2, CYP21, CYP11B2 cascade is hypothesized to lead to increased aldosterone secretion, expanded plasma volume, and hypertension. Furthermore, *UBP1* is also expressed in mouse heart and aorta [28] (GEO profiles, www.ncbi.nlm.nih.gov/sites/entrez). However, the role of CYP11A, 3β-HSD2, CYP21 and CYP11B2 in human heart and aorta is a matter of debate [29],[30]. If UBP1 could also transactivate the *CYP11A* promoter in heart and aorta, UBP1 might drive the cardiac remodeling via control of aldosterone levels. The closest *UBP1* gene homologue, *TFCP2L*1 (also known as *LBP-9*), suppresses *CYP11A* transcriptional activity in placental cells [27] and *LBP-9* gene-trapped mice die postnatally with a dramatically decreased expression of kidney salt handling genes or survive with a mild polyuria and an increased net electrolyte excretion [31]. The antagonistic actions of LBP-9 and UBP1 on the *CYP11A* expression suggests that these two genes could play a counterbalancing role in blood pressure control. Approaches aimed at modulation of *UBP1* activity and targeting the *UBP1* driven genes could represent novel therapeutic approaches to treat hypertension and eventually other cardiovascular diseases.

## Methods

### Blood pressure measurements in the mouse

All animal work was conducted according to French and EU guidelines. The BXD advanced recombinant inbred mouse strains were generated by crossing C57BL/6J and DBA/2J mice, intercrossing progeny for ten generations with intentional avoidance of sib mating, and finally inbreeding the advance intercross stock for more than 20 generations [32]. All mice were 16-weeks-old and were fed standard rodent chow when phenotyped. The systolic BP was measured by a computerized tail-cuff system (BP-2000, Visitech Systems, Apex, NC) in conscious animals. Following 10 preliminary measurements in pre-warmed tail-cuff (36°C) device to accustom mice to the procedure, 10 actual measurement cycles were collected on 5 consecutive days at fixed diurnal interval and averaged for each individual animal. As movement artefact could reduce the number of successful recording, the daily procedure was considered successful when 7 out of 10 measurements were valid with a standard deviation less than 10 mmHg. BP recording was performed on 5 consecutive days. For each individual, BP mean of 5 consecutive days was averaged and used for analysis. The BP was determined using n = 1 (10% of BXD strains), n = 2 (70%) and n = 4 (20%) of mice per BXD strain.

### EUGENE2 genome-wide association study

#### Participants

The participants included in this study were healthy, non-diabetic offspring of patients with type 2 diabetes, as previously described in detail [20]. A total of 466 families were included (number of offspring from the families were: one in 265 families, two in 106 families, three in 54 families, and four or more in 41 families). One of the parents had to have type 2 diabetes and the other parent normal glucose tolerance in an oral glucose tolerance test and/or no history of type 2 diabetes in the family. The probands were randomly selected among type 2 diabetic patients living in the regions of five centers in Europe. They were recruited over a 4-year period through advertisements in public media and in the hospitals. The acceptance rate of volunteers was at least 70% in the different centers. Altogether 869 offspring, having blood pressure measurements, were included in the study from the following centers: Copenhagen, Denmark (n = 257), Gothenburg, Sweden (n = 134), Kuopio, Finland (n = 299) and Tubingen, Germany (n = 179). The appropriate Institutional Review Boards approved the study protocol. All study participants gave informed consent.

#### Measurements

All centers followed the same protocol. BP was measured with a mercury sphygmomanometer in the sitting position after a 5 min rest. The average of two measurements was used in statistical analyses. BP of individuals who were on BP medication (8.6% of study subjects) was adjusted by adding 15 mmHg to systolic and 10 mmHg to diastolic BP [33]. Height and weight were measured to the nearest 0.5 cm and 0.1 kg, respectively and body mass index (BMI, kg/m2) calculated. Because the primary aim of the EUGENE2 Study was to investigate the genes associated with insulin secretion and insulin sensitivity every participant underwent detailed metabolic studies, as described [20].

#### Sample preparation, genotyping, and quality control

The GWA analysis was carried out in the Finnish Genome Center in Helsinki. A single nucleotide polymorphism (SNP) genotyping was performed with the commercial release of the Infinium HumanHap 550 k version 3 chips (Illumina, San Diego, CA), containing SNPs derived from Phase I and II of the International HapMap project. Briefly, 750 ng of DNA was used in genotyping according to the manufacturer's protocol (Illumina). Software package PLINK (http://pngu.mgh.harvard.edu/~purcell/plink/) [34] was used for quality control and association analysis of the GWA data.

#### Individual exclusion criteria

Gender calls from X chromosome genotype data was verified to be in concordance with the reported gender of each individual. To verify known familial relationships and to detect not reported first-degree cryptic relationships, pairwise identity-by-descent analysis was performed. Individuals with genotyping call rates less than 95% were excluded, resulting in total of 903 out of 970 samples passing these criteria. Of these individuals, 869 had the required phenotype data and these individuals advanced to the actual association analysis. Total genotyping call rate in remaining individuals was 99.6%.

Complete linkage agglomerative clustering based on pairwise identity-by-state (IBS) distance in addition to multidimensional scaling plots were used to assess population stratification in the data. Concordance between study center and IBS distances was observed, and therefore the study center was included in all statistical models to adjust for population stratification.

#### Marker exclusion criteria

Markers advanced to the actual association analysis if they passed the following quality control criteria 1) had a 95% genotype call rate (4007 markers excluded), 2) had a minor allelic frequency (MAF) >1% (23107 markers excluded) and 3) demonstrated Hardy-Weinberg Equilibrium with a P>1e-05 (890 markers excluded). Total of 534 287 markers passed these quality control criteria. In addition, for each marker, tests for MAF, Hardy-Weinberg Equilibrium and missingness were performed, and this information was used when evaluating marker quality for replication.

#### Marker imputation

We used Phase 2 HapMap (release 23) CEU founder population data (60 individuals with MAF>0.01 and genotyping rate >0.95) to impute markers that were not directly genotyped. PLINK's discrete genotype calls imputation with confidence threshold of 0.8 was used for imputation. Same marker exclusion criteria that were used for filtering directly genotyped markers were used for imputed markers.

Initial marker screening was directed to the genomic location indicated by the mouse model (human chromosome 3, 33.2–33.9 Mb region). After marker exclusion there were 306 markers located on this region (76 directly genotyped, 230 imputed).

#### Marker selection for replication studies

We selected the SNPs for replication on the basis of the most significant *P* values in the EUGENE2 GWAS (9 SNPs were selected, arbitrary cut-off point of *P*<0.0034 in the EUGENE2 Study). Furthermore, the genotype distribution of these SNPs had to follow Hardy-Weinberg expectations, and their MAF was >5%.

### METSIM replication study

#### Subjects and methods

METSIM (METabolic Syndrome In Men) Study is an ongoing study aiming to include 10 000 men, aged from 50 to 70 years, randomly selected from the population register of Kuopio town, Eastern Finland (population of 95 000) [21]. Every participating subject has one-day outpatient visit to the University of Kuopio, including an interview on the history of previous diseases and current drug treatment, and an evaluation of cardiovascular risk factors. Blood pressure (BP) was measured in a subject's sitting position after a 5-minute rest with a mercury sphygmomanometer. The average of 3 measurements was used to calculate systolic and diastolic BPs. Height and weight were measured to the nearest 0.5 cm and 0.1 kg, respectively. Body mass index was calculated as weight (kg) divided by square of the height (m). The study protocol was accepted by the Ethics Committee of the University of Kuopio and Kuopio University Hospital.

#### Genotyping

Genotyping was performed using the TaqMan Allelic Discrimination Assays (Applied Biosystems) or using the iPLEX Sequenom MassARRAY platform.

### RIAD replication study

#### Subjects and methods

A total of 622 (284 men and 338 women) participants of the RIAD Study, a prospective survey on the *R*isk factors in *I*mpaired glucose tolerance for *A*therosclerosis and *D*iabetes, were included in analysis [22]. In brief, subjects from 40–70 years of age were examined for those who had risk factors for the development of T2DM, such as a family history of T2DM, obesity, and/or hyper/dyslipoproteinemia. Known diabetes, medication affecting glucose tolerance, liver and kidney diseases, thyroid gland functional disorders, and acute infections represented exclusion criteria. Written consent was obtained from all participants. Subjects aged ≤55 years were included in the study (N = 237). The study protocol included measurements of BP, weight, height, and waist circumstance. Systolic and diastolic BPs were measured in a subject's sitting position after a 5-minute rest with a mercury sphygmomanometer. The study was accepted by the local Ethics Committee.

#### Genotyping

Genotyping was performed using the TaqMan Allelic Discrimination Assays (Applied Biosystems).

### Association analysis of genotyping results

Association analyses of the genotyping results were carried out using SPSS 15.0 for Windows (Chicago, IL, USA). Association was tested for both systolic and diastolic blood pressures and variables with skewed distribution were logarithmically transformed for statistical analysis. For analysis of EUGENE2 genotyping results, mixed linear models were used. We included the family (sibship) and centre as random factors, the sex as a fixed factor, and both age and BMI as covariates. For analysis of the METSIM and RIAD genotyping results, linear regression models were used, and both systolic and diastolic blood pressures were adjusted for significant covariates (age and BMI for METSIM, and age, BMI and gender for RIAD). Additive model was used for all analysis. Effect sizes were calculated in all of the studies by using linear regression, with non-log transformed BPs as dependent variables. BPs were adjusted for significant covariates. For both traits the pooled test of association was determined using fixed model inverse variance weighted meta-analysis from R's gap: Genetic analysis package version 1.0-17 (http://cran.r-project.org/web/packages/gap/).
